# Social and ecological conditions of cranberry production and climate change attitudes in New England

**DOI:** 10.1371/journal.pone.0207237

**Published:** 2018-12-12

**Authors:** Brian J. Gareau, Xiaorui Huang, Tara Pisani Gareau

**Affiliations:** 1 Department of Sociology, Boston College, Chestnut Hill, Massachusetts, United States of America; 2 Earth and Environmental Sciences, Boston College, Chestnut Hill, Massachusetts, United States of America; DePaul University, UNITED STATES

## Abstract

Cranberry growers in New England are increasingly pressured by negative effects associated with global climate change, some of which are familiar to this group (such as precipitation fluctuations and pest pressures), others that are rather new (such as warmer winters that threaten needed chill hours for the plants to bloom). The first study of this population of its kind, we use a survey, supplemented with observations and interviews, to assess Massachusetts cranberry grower attitudes towards climate change, and whether certain conditions of production might be associated with their attitudes. Our findings suggest that certain personal and ecological conditions are associated with greater worry of climate change effects, and that communal conditions of the cranberry grower social network provide some ways to cope with a warming climate. While the cranberry growing community has created a strong social network that has allowed it to sustain production, a warming planet will likely require significant change in order to overcome general attitudes of climate skepticism so that cranberry production may continue in the future.

## Introduction: Climate change and farmer attitudes in the United States

**Fig 1:**
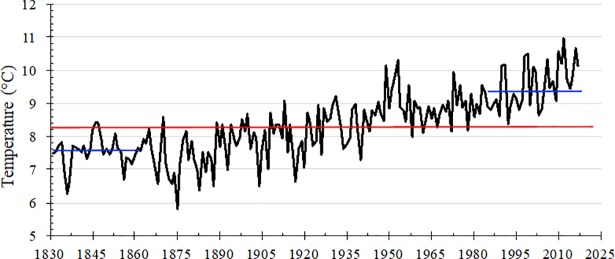
Mean annual temperature (1831–2017). Temperatures measurements are from Blue Hill Observatory in Milton, MA. The red line represents the average temperature (8.32°C) across the time period. The blue lines represent the mean temperature during the first 30 years of the record (7.59°C) and the mean temperature during the last 30 years of the record (9.81°C).

Surface air temperature has increased in the Northeastern United States at a faster rate than the global trend of about 1°C since 1880 [1]. According to Blue Hill Observatory, one of the oldest weather stations in the United States and 30 miles south of Boston, Massachusetts, the mean annual temperature from the most recent 30-yr period of 1988 to 2017 was 9.6°C, which is 1.9°C warmer than the mean temperature between 1881 and 1910 of 7.7°C, and 2.08°C warmer than the 30-year average between 1831 and 1860 of 7.5°C (Fig 1). It is projected that the Northeastern United States will experience between 2 and 5°C of additional warming by the end of this century [1]. This accelerated warming is especially threatening to perennial temperate crops that are adapted to cold temperatures. Unlike other perennial crops, cranberries have a narrow niche breadth, requiring a specific set of ecological conditions that must be met for adequate yields to be achieved and to avoid crop failure. Additionally, cranberry growers necessarily establish an intimate relationship with their surrounding landscapes due to their dependency on various natural resources, such as large quantities of water at multiples times of the year and pollinating insects [2]. Associated effects of global warming, such as extended droughts, increased precipitation in shorter time periods, and an increase in pressure from insect, weed, and disease pests, further challenge the sustainability of cranberry production.

The cranberry industry is of great social, historical and ecological significance to Massachusetts, being intimately tied to the development of the state since the days of the Pilgrims, associated with one of its most historic regions, Cape Cod, and valued by the community as an integral component of the Massachusetts landscape. Our study investigates the attitudes that cranberry growers in Massachusetts hold towards global climate change and its effects on their own livelihoods. In order to understand cranberry grower attitudes towards climate change, it is necessary to understand the broader social and ecological context in which cranberry growing is embedded. In this way, cranberries stand out as a unique agro-ecological system, one that makes neat divisions between agriculture and the surrounding landscape impossible to create. It is useful, then, to draw from theories in environmental sociology, a discipline that has long engaged with society-nature relations in efforts to discover how various social groups interact with their surroundings in socially, politically, economically, and ecologically entangled ways [3,4].

In the United States (US), agricultural systems are among the most vulnerable economic sectors to climate change [5–7]. While increases in atmospheric CO_2_ and lengthening of the growing season has generally increased plant productivity, including for several important grain crops [8], throughout much of the US in the 20th century [9], drought and extreme temperatures occurring during critical growth periods relate to higher yield variability for annual crops [10–13]. The negative impacts of climate change on agriculture are expected to increase over time [14–16]. Yet, the effects and impacts of climate change are heterogeneous across the US [1]. For instance, the frost free season has lengthened by 2 to 3 weeks in the Western US, 1 to 2 weeks in the Northeast and Midwest, and less than 1 week in the Southeast [6]. A longer growing season in the Northeast and Midwest can have the positive effect of increasing opportunities to grow subtropical and tropical crops for those growers willing to try new crops. Yet grain crops tend to perform worse under a warming trend especially under extreme heat during pollination and grain filling [13]. Multiple simulations from a dynamic ecosystem model predict a 6 percent decrease in Iowa maize yield in the 21st century for every 1°C rise in warm season average temperature [17]. Yield models with low and high emissions scenarios predict declining yields for California’s maize, wheat, cotton, and sunflower and slightly increased yields for alfalfa [14]. Perennial temperate crops that require a period of winter dormancy, known as chilling hours or vernalization, are particularly vulnerable to temperature increases in the winter.

Due to variability in the effects of climate change, scholars point to the need to understand the local context of agriculture so that adaptation efforts may be tailored to the specific social and ecological circumstances of agricultural systems [18,19]. The call to focus on the local context is not limited to agriculture, but rather is found in a range of literatures recognizing local sites where climate change will be engaged with in meaningful ways due to the ways in which ecological conditions become entangled in social, political, and economic conditions on the ground in locally-specific arrangements [3]. As actors operating in agriculture, growers acquire an intimate understanding of their landscape, yet that understanding does not always translate into attitudes and action that form links between local and global systems. Studies of relevance to this paper engaging in this terrain range from: 1) comparisons between (and deviations from) local and regional observations of climate change and the global consensus on its effects (for a thorough review of nearly one hundred studies in this area, see [18]; see also [20]); to 2) climate change beliefs and the perceived risks to specific agricultural (and other) systems among stakeholders (Cf. [21–26]).

Regarding views of climate change and the perceived risks to agriculture, Chatrchyan et al. [22] provide a comprehensive review of the literature from within the US context. The authors find that farmers’ attitudes towards climate change, and its anthropogenic nature, vary across studies based on region and agricultural system. The findings suggest that “farmers relate to climate change based on their own experiences with its impacts,” but that views about its anthropogenic nature are harder to link to causal explanations, though are likely connected to farmer’s predominantly conservative political views [22]. Studies from around the US rather consistently report that American farmers hold conservative political views that likely influence their attitude towards climate change, leading to a disconnect between local experience of climatic changes and ‘global knowledge’ of global climate change as reported by the scientific community (See [22] for a comprehensive review, and [7] for a recent analysis of this phenomenon among Idaho farmers).

Other studies discover variability in farmer perceptions of climate change across regions, crops, and other factors, such as length of experience with farming. For instance, Diggs [27] found significant difference in belief that climate change is occurring between farmers surveyed in Colorado and North Dakota, with the former being more skeptical, but also that those with less farming experience are more likely to believe that negative events such as extreme drought were becoming more severe. Safi et al. [28] discovered that over sixty percent of surveyed farmers in Nevada believe climate change is taking place, but less than thirty percent believe it is due to human activity or that it is having an effect on current adverse environmental conditions. Schattman et al. [29] reported that Vermont farmers have some of the highest reported beliefs that climate change is occurring among studies conducted nationwide (eighty percent), whereas Marchant and Bosc [30] report some of the lowest levels of belief in Mississippi (under twenty-five percent). (For thorough reviews of these and more studies on farmer attitudes towards climate change, see [22,25]). Arbuckle et al. [21] state close to seventy percent of Iowa farmers believe in climate change, but less agree that it will have any sort of impact on their own farm operations (thirty-five percent). Such studies reveal the range in attitudes towards the reality of climatic change among farmers, as well as its impacts on their own production platforms.

We know from studies in the social sciences on climate change attitudes among the general population that education has an effect on changing beliefs of climate change that would counter their political ideology, yet the effect is reduced, if not reversed, among white educated males (Cf. [31,32]). Yet, the agricultural community–especially extension agents and others operating in the land grant system–are often working to find ways to connect local experiences with climatic change to mitigation and adaptation strategies through education, even among growers who are primarily white males. As people making a living through agriculture, extension agents in some cases work to tap into the “experiential learning” that some growers and farmers in their region have received as climatic changes effects become increasingly difficult to ignore, leading to some farmers making connections to the reality of global warming [33]. Here, strategies that have been the most successful take the local context into account so that the proposed changes to agricultural systems make sense given the specific conditions of the farmers who need to implement the change, both for their own sustainability as growers and for the sake of the planet [22,34].

Below, we use a social theoretical approach to help us comprehend grower attitudes in their broader context. Data were gathered with a survey distributed to cranberry growers in Massachusetts, supplemented (less centrally in this paper) with observations of cranberry production at multiple research site visits as well as in-depth and informal interviews. Specifically, we draw from O’Connor’s theorization of the *personal*, *communal and ecological conditions of production* to frame the positioning of cranberry growers–and the attitudes they hold towards climate change–in their local and regional contexts [35,36]. This frame allows us to take seriously the attitudes that growers hold vis-a-vis their livelihoods, access to resources, and other production conditions. Investigating conditions both on and off the cranberry operation proper helps us discern how specific cranberry growing conditions in one context might differ from those of another, thus contributing to differences in attitudes towards global climatic change.

## Personal, communal and ecological conditions of cranberry production

Drawing from Karl Polanyi, O’Connor [35] posits that three conditions are necessary for a capitalist economy to function, including the agricultural sector. Polanyi defined labor as a “fictitious commodity,” meaning it was a necessary component of the economy, but if it were used strictly as such, it would perish. Drawing from this definition, O’Connor explains that labor is a “personal condition” in that it is a person’s labor power that can be accessed by the market, and that access depends upon whether the person is willing and able to work for a given wage under certain conditions [35]. In the case of cranberry production, most of the labor is completed by the cranberry grower and his or her family members, with seasonal work sometimes conducted with the assistance of hired hands to help with the harvest. The value of the harvest, the cost of owning land, and other factors affect how much growers can pay hired hands, and it also determines how much profit they can make themselves. We know that, nationally, median income from farm activities is below the breakeven point, such that most farm households depend primarily on off-farm income. Hence farming conditions are economically precarious or unfruitful for many in the industry (https://www.ers.usda.gov/topics/rural-economy-population/), meaning growers are willing to work in the industry for lifestyle choice, itself a personal condition of production (see also [37–39]).

We would emphasize in O’Connor’s personal conditions a need to focus on the age, gender, and education level of the cranberry growers, because these personal conditions (some quite biological, such as age, others social, such as gender and education) have an impact on how one approaches the labor process, and likely reveal how various actors view their condition vis-a-vis the broader social and economic landscape. Socialization impacts individuals differently, and we know from previous studies that age, gender, and education have an impact on attitudes towards society-ecology interactions such as those caused by climate change [40]. Therefore, personal conditions necessarily overlap with communal and ecological conditions in disparate ways that have diverse effects.

Communal conditions of production are the social and physical infrastructures required for economies to operate, but oftentimes are not a part of specific production platforms [35]. Roads, communication, education systems, and extension networks would all be considered communal conditions that allow cranberry production to happen. Importantly, O’Connor points to the cultural dimensions of communal conditions, where society reproduces the conditions necessary for certain economic activities to take place. The importance of cranberries in one of America’s most symbolic and celebrated holidays (Thanksgiving), the cultural importance of cranberries to the development of Massachusetts (e.g. drawing several hundred thousand tourists worldwide to the annual Cranberry Harvest Festival, held in Wareham, MA), and the value that policymakers and local citizens place on the importance of agricultural land (cranberry bogs in this case) for a thriving social and ecological landscape are all outside the realm of cranberry production but part of what allows it to persist (see also [39]). Communal conditions provide the social framework in which actors operate, where norms of sharing advice, equipment, access to water in adjacent wetlands, and other resources can be created to allow livelihoods to persist even in difficult situations. Therefore, we can consider farmers’ attitudes towards climate change as being constituted partly by the cultural fabric of the cranberry community. Asking questions, then, about global warming attitudes is to interrogate a facet of the communal conditions of production.

O’Connor calls the third condition of production the “natural condition,” meaning those environmental conditions “external” to the economy but necessary for it to function. Soil quality, abundance of water, and all the processes that the natural world provides “outside” of the economy would be considered among these conditions. Natural conditions are considered “external” to the economy until they become recognized as having value and thus become factored into the production process [41]. Recently, however, scholars have referred to these as *ecological* conditions, or *socionatural* conditions, in order to bring out the mutually constitutive, hybrid character of social and natural systems [3,42–45]. Whether factored into production economically or otherwise, the natural world is a dynamic part of the production system, and its condition can either help or hamper the economy based on the health and sustainability of the relationship. In cranberry production, the surrounding ecological landscape, and the weather that shaped it, has an important role to play, either providing the grower with an abundance of inputs such as favorable weather conditions, adequate water, and pollinating insects, or containing a poorer composite of these factors (see below). Such conditions are not factored into the value of cranberries on a site-by-site basis, but they play an intricate role in the entire production process. The geographies of particular cranberry systems vary across systems, as does ownership of the ecological resources that are a part of them. Therefore, it is useful to think of ecological conditions as contributing to the cranberry production system in a state of mutual cooperation or friction, and the dynamic makeup of those conditions and one’s ability to access them have an impact on that relationship.

What is of particular novelty in this paper is our interrogation of cranberry growers’ relationship with ecological conditions of production. How growers have interacted with specific weather patterns and other conditions that affect cranberry harvests over the long-term likely has an effect on how growers interpret climate change. O’Connor [35] provides another factor to consider: ownership of ecological conditions. For cranberry growers, personal conditions influence how one approaches their cranberry operation, but access to ecological conditions is also important, especially access to an abundance of water, as we will see below. Communal conditions, which include the rights that society (through the state) grants cranberry growers to use protected areas for their water, is also important, as is the cultural importance that society places on cranberry bogs in the landscape, and the sense of sharing that goes on between growers. We would expect that having direct ownership of these natural resources would have an effect on growers’ perceptions of their own vulnerability to climate change, because ownership provides immediate, unequivocal access to resources whenever needed. Studies on the political ecology of inhabited protected areas suggest that ownership of resources and land has an impact on how people engage with issues of resilience and perceptions of the natural environment (Cf. [46–48]). However, with ecological conditions being varied, dynamic, and fragmented, it is difficult to hypothesize which specific ecological conditions will be more significant than others.

### Ecological conditions of cranberry production

The American cranberry, *Vaccinium macrocarpon*, is a temperate perennial wetland plant native to northeastern North America. Native cranberries can still be found in moist low lying areas that naturally flood. Commercial cranberry bogs were first established in Massachusetts in the 1800s in peat-filled, impermeable kettle holes formed from glacial deposits. Many of the original kettle bogs are still in production today [49]. However, modern bogs (post 1986) are dug in upland habitat soil and made to replicate a wetland system.

**Fig 2:**
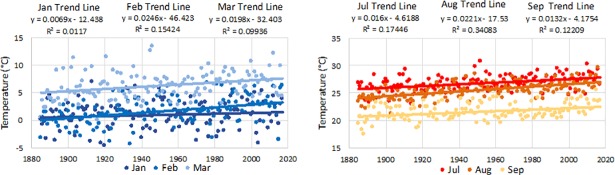
Monthly mean maximum temperature in winter months and summer months (1885–2017). Temperatures measurements are from Blue Hill Observatory (Milton, MA), the oldest continuous weather record in North America and within 50 miles from the UMass Cranberry Experimental Station.

Cranberries are distinctive from other cropping systems in a number of ways. First, cranberries require between 1,000 and 2,500 chill hours (accumulated hours between 0°C (32^∘^F) and 7.2°C (45^∘^F) depending on the variety, which is more than other temperate fruit crops. This is an important distinction, because in New England the average monthly high temperature is rising across winter months and at a faster rate and with higher variability than in summer months (Fig 2). Accumulating sufficient chill hours allows the cranberry plants to break dormancy and begin to develop once temperature and length-of-day are favorable. When winter temperatures rise above 7.2°C (45°F) for an extended time, there is a risk that cranberry bud development will be impaired. Dormant mixed-terminal buds (buds that will bloom next year) fail to develop when exposed to less than 1,000 chill hours, and the more chill hours accumulated above the 1,000 requirement the shorter the amount of days to bud break [50].

Second, there are no crop rows or furrows in a cranberry bog; the soil is completely covered with vines forming a thick mat that extends over the entire bog. The perennial cover that cranberries provide conserves soil and prevents the establishment of annual weeds, but perennial weeds that spread in the cranberry understory and underground via stolons and rhizomes, can significantly depress cranberry yields [2]. Third, cranberry bogs are highly dependent on freshwater coming from either ponds or groundwater. The bogs are seasonally flooded in the fall for harvest, in the winter for protection, and in the spring for pest management. Flood water is controlled with a system of ditches, dykes, and gates that allow the water to flow from a bog of higher elevation to another of lower elevation. Sharing water for seasonal flooding is a typical cultural practice among cranberry growers, a communal condition that has allowed production to be sustained in the region. Fourth, cranberries are grown as a monoculture; the acidic soil conditions, growth habit of the cranberry plant, and periodic flooding, limits the opportunities for intercropping and crop rotation. Although, fifth, intra-specific genetic diversity can be high with several varieties of cranberries, including ones from the 1800s, growing in a single bog. Sixth, and important for climate mitigation, cranberry bogs likely sequester more carbon than they release due to zero tillage, the periodic practice of sanding, which buries the cranberry vines (organic carbon) to stimulate new growth and buds, and the conservation of carbon-rich peat in the peat-based bogs.

Finally, cranberry bogs are distinctive in that they are embedded in a larger network of forests, ponds, and streams. Growers depend on natural ponds to flood or irrigate their bogs and forests and wetlands for sources of wild bees important in pollination. For every acre of managed cranberry bog, growers typically manage several more acres of upland and wetland habitat [2]. Thus cranberry bogs, while monocultures themselves, are part of a diverse ecological landscape that conserves many plant, bird, mammal, reptile, amphibian, arthropod, and fish species.

To investigate with a finer grain the links between conditions of cranberry production and attitudes towards climate change, we have devised the following research questions:

1. What are the general attitudes towards global warming among cranberry growers, and how do they compare to the American public in general?
2. What are the effects of specific personal, communal, and ecological conditions of cranberry production on global warming attitudes?

## Methods

### Survey

In January 2016, a solicitation to participate in our ninety-seven question survey was disseminated to all the approximately four hundred cranberry growers of Massachusetts with contact information held by either the University of Massachusetts Cranberry Station, and/or the Cape Cod Cranberry Growers Association (CCCGA). The U-Mass Cranberry Station emailed the solicitation to its list of growers, and the CCCGA posted an advertisement to participate in their monthly newsletter, *Bogside*, throughout the 2016 year. Subsequently, the researchers mailed a hard copy of the solicitation, along with the survey and postage-paid return envelopes, to all growers on the CCCGA mailing list who had not returned the survey (The names of these members, and their contact information, was not released to the researchers in order to maintain CCCGA membership privacy). Online respondents read a consent form explaining the nature of the study prior to answering questions, and respondents to the mailed survey were given a hard copy to review prior to answering the questions. This research complies with ethical practices and was approved by the Boston College Institutional Review Board (Protocol number 12.139.01e) prior to disseminating the survey. Survey respondents were assured that their identities would be kept anonymous in any publications using these data. We received ninety-one responses out of roughly four hundred cranberry growers, approximately twenty-three percent of the grower population (Table 1). For a population that is much less varied than the general public (predominantly Caucasian, all from the same region in Massachusetts), this is an adequate sample size with between a ± 5 percent and ± 10 percent sampling error at the 95 percent confidence level [51]. Our sample reflects a large portion of total cranberry acreage in Massachusetts (over forty percent), and roughly twenty percent of upland habitat owned by cranberry growers (Table 1). Therefore, the sample is highly suitable to ask questions about ecological conditions of production, as it represents a sizable portion of the cranberry community and production area.

**Table 1:** Sample size and representativeness.

	Total in the sample	Total in MA	% Represented in the Sample
Number of Growers	91	around 400	23%
Acres of Cranberry Bogs	5,870	around 14000	42%
Acres of Upland Habitat	10,129	around 48000	21%
Acres of Wetland Habitat	4600.05	n.a.	n.a.

Source of acreage in MA: Cape Cod Cranberry Growers Association website: https://www.cranberries.org

### Supplementary: Observations and interviews

Our survey responses are informed by observations while gathering ecological data over the past eight years at bog sites in the region, as well as formal and informal interviews with cranberry growers. Interview data are limited here, but will be central to future publications. One of the authors (Pisani Gareau) has been gathering ecological data, from 2015 –present, on bog-insect-weed interactions in a warming climate that are part of the authors’ larger interdisciplinary research project. This work has led to multiple conversations with dozens of cranberry growers that have helped us discern our survey results and draw conclusions from the findings. Two authors (Pisani Gareau and Gareau), along with research team members, have conducted twenty interviews between 2012 and 2018 with cranberry growers to learn more about the history of cranberry production, as well as grower attitudes towards environmental conditions, including climate change. These interviews helped us make sense of survey results as well, contributing to our understanding of personal and communal conditions of cranberry production in particular.

#### Hypotheses

Based on the extant literature, our observations in the field, and our general understanding of cranberry growers and their conditions, the following hypotheses emerged that we tested with statistical analysis of the survey data:

- Growers reporting that ecological conditions of cranberry production are worsening will place greater importance on global warming [21], and in general will express more concern than the American public.
- Specific reported ecological conditions, such as water availability, increasing temperatures, pest pressures, etc. will have more of an effect on global warming attitudes because cranberry bogs are embedded in a diverse landscape and highly dependent on water resources, appropriate temperatures for bud and fruit development, and pest management. Thus we would expect that growers who report greater pest pressure, will see global warming as a greater threat. Similarly, growers who report low access to water resources, will be more likely to view global warming as a threat or to worry about it.
- Increased ownership of ecological conditions of production, especially water but also dry upland habitat, will have an inverse effect on attitudes towards the importance of global warming, because increased access to these ecological conditions of production buffer growers from the negative effects of global warming. The novelty of cranberry production might explain why this hypothesis has not previously been tested in other studies.
- Personal production conditions such as gender, education, and age will have an effect on attitudes about the importance of global warming, with women, higher education individuals, and younger individuals being more aware of climate change risks than men, less educated and older individuals [30,40].

## Data analysis

### Dependent variables: Global warming attitudes

We model two dependent variables on attitudes toward global warming. The first variable, used as well by McCright, Dunlap, and Xiao [52] *global warming threat*, measures the binary responses to the survey item “Do you think that global warming will pose a serious threat to you or your way of life in your lifetime?” (1 = yes, 0 = no). Cranberry growers have an intimate connection to their surrounding landscape, including its weather, and so we expect they can identify long-term changes that might escape other actors, especially among conservative populations. Thus, we expect that cranberry growers in general will view global warming as a threat in their lifetime. Since growers report being more skeptical of global warming in general, it is possible that some connections to the landscape have an impact on attitudes. The second dependent variable, also examined by Dunlap, McCright, and Yarosh [53] and Chong [54] in terms of political division, *global warming worry*, measures ordinal responses to the survey item “Please tell us how much you personally worry about global warming?” (a 1 to 4 scale: “not worry at all” to “worry a great deal”). Given the socio-ecological relationship between cranberry growers and ecological conditions of production (chill hour requirements, water availability, pollinators, etc.), we anticipate that Massachusetts cranberry growers are more concerned about global warming than the average American.

### Independent variables: Ecological conditions of production

We have three groups of key independent variables. Group One measures general conditions related to cranberry growing and contains 3 variables. *General conditions* is a dummy variable that measures responses to the survey item “Over the past 5 to 10 years, how are conditions in general for cranberry growers?” (1 = getting worse, 0 = remaining the same or getting better). In the questionnaire, we provided 3 response categories: getting worse, remaining the same, and getting better. In the analyses, we combined the categories of remaining the same and getting better because only 5.5% of respondents chose getting better. It is impossible to know precisely what types of conditions growers referred to when they answered this survey question. Nevertheless we argue that it is acceptable to assume that this question primarily reflects growers' perceptions of an assortment of environmental conditions, and to a lesser extent, their perceptions of other, non-environmental conditions of cranberry production. This is because, first, we positioned this survey question amongst questions on specific environmental pressures/conditions. Thus, growers may have thought primarily of environmental conditions when they answered this question. Second, we calculated the bivariate correlations between the dummy variable on general condition and a series of variables measuring growers' perceptions of the severity of environmental pressure (12 variables) and non-environmental pressures (8 variables). The results show that a group of 5 variables on environmental pressures and only 1 variable on non-environmental pressures (i.e. the concern over price of cranberry) are significantly (a = 0.10) albeit weakly correlated with the variable on general conditions. None of the other variables examined are significantly correlated with the variable on general conditions and the magnitude of the correlations are weak. *Weather importance* measures responses to the survey item “Have weather-related issues become more important (worse) over the past 5 to 10 years?” (1 to 5 scale: not important to very important). *Weather counts* records the number of responses from each cranberry grower to the survey item “which of the following specific weather-related events severely impacted your cranberry production from 2010- present? Check all that apply.” Respondents chose from 6 answer categories: high precipitation in short amount of time, drought, summer heat wave (temperature above 90 for several days), warmer winter, hail or sudden frost event, and other. Respondents who chose “other” were invited to elaborate on their responses in the questionnaire; and all these respondents wrote salt-water incursion as their elaboration.

Group Two measures specific ecological conditions that are essential to cranberry production (these conditions are discussed in detail above). Respondents were asked to rate the importance of 9 specific aspects of ecological conditions observed in the last 5 to 10 years: 1) water scarcity for irrigation, 2) competition for water, 3) heavy precipitation, 4) extended high temperatures in the summer, 5) extended high temperatures in the fall, 6) insufficient chill hours in the winter, 7) insect pests, 8) weed pests, and 9) diseases. The responses are on a scale from 1 to 5 with 1 being not important and 5 being very important.

Group Three measures cranberry growers’ access to important ecological conditions/natural resources. Upland habitat acreage and wetland habitat acreage are self-reported sizes of upland habitat (meadow, forest, etc.) and wetland habitat (swamps, dug ponds, etc.) within each respondent’s farm. As discussed above, upland and wetland habitat ownership are important ecological conditions of production due to the access to water they provide, as well as habitat to pollinators and other beneficial insects. Water is a critical component to cranberry production throughout the year, and growers have varying levels of access to it. Acres of upland habitat, wetland habitat, and cranberry bog are measured in tens of acres and are top-coded to correct for outliers with extremely large values. We also include a variable that measures the relative size of natural habitat (of both types) as percentage of total farm size.

### Independent variables: Personal conditions

We include educational attainment, age group, and gender of cranberry growers as independent variables of *personal conditions* of the growers. All these personal conditions of production are found by prior studies in agriculture and the general American public to be predictors of climate change attitudes [7,20,32,55]. We do not explicitly control for political ideology or party affiliation because we know from our years of conducting research on cranberries that this is a sensitive question that can be disruptive to researcher/grower collaboration. However, we did ask questions about how climate change is portrayed in the media as a proxy for political orientation, as past studies have shown that people with conservative political affiliations are more skeptical about media coverage of global warming. Descriptive statistics of the overall sample are provided in Table 2.

**Table 2:** Descriptive statistics of the overall sample.

Variable	Description	N	Mean	Std. Dev.	Min	Max
**Climate change attitudes**						
Global warming threat	Whether respondents think that global warming poses a serious threat to themselves or their ways of life in their lifetime. Yes = 1, no = 0	89	0.42	0.50	0	1
Global warming worry	How much respondents worry about global warming, 1–4 scale from *not worry at all* to *worry a great deal*.	90	2.21	0.89	1	4
**General environmental conditions**						
General conditions	Changes in the general conditions for cranberry growers over the past 5–10 years. 1 = getting worse, 0 = not getting worse.	89	0.70	0.46	0	1
Weather importance	Rate the importance of weather-related events in the past 5–10 years, 1–5 scale from *not important* to *very important*	87	3.82	1.04	1	5
Weather counts	Count measure of specific types of weather-related events severely impacted cranberry production from 2010-present	91	2.12	1.27	0	5
**Specific environmental conditions**						
Water scarcity	Rate the importance of these environmental conditions in the past 5–10 years, 1–5 scale from not important to very important	91	3.65	1.38	1	5
Competition for Water		90	2.99	1.43	1	5
Heavy Precipitation		91	3.54	1.27	1	5
High Temperatures in Summer		89	4.02	0.92	2	5
High Temperatures in Fall		90	3.56	1.03	1	5
Insufficient Chill Hours in Winter		87	2.98	1.10	1	5
Insect Pests		90	4.06	0.90	2	5
Weed Pests		90	4.11	0.83	2	5
Disease		90	4.03	0.92	2	5
**Access to natural resources**						
Wetland habitat acreage	Acreage of wetland habitats, top-coded. Unit: 10 acres	85	3.94	7.58	0	30
Upland habitat acreage	Acreage of upland habitats, top-coded. Unit: 10 acres	88	10.26	17.44	0	80
Relative size of natural habitats	Size of natural habitats as percentage of total farm size	83	67.05	19.12	0	98.40
**Control variables**						
Educational attainment	1–9 scale from *No schooling completed* to *Doctorate/Professional degree*	89	6.03	1.39	4	9
Age category	1–6 scale from *25–34 y*.*o*. to *> = 75 y*.*o*.	90	4.18	1.16	1	6
Female	Being female. Yes = 1, no = 0	88	0.24	0.43	0	1
Cranberry acreage	Acreage of cranberry bogs, top-coded. Unit: 10 acres	91	4.07	5.27	0.1	20

For the binary outcome *global warming threat*, we estimate logistic regression models. For the ordinal outcome *global warming worry*, we estimate generalized ordered logit regression models. This modeling technique tests for each independent variable if the proportional odds assumption is violated. If it is violated for an independent variable, the estimator generates multiple coefficients, each coefficient corresponding to a cut point in the ordinal outcome. Otherwise, the estimator generates one coefficient for each independent variable. This modeling technique is less restrictive than the proportional odds models but more parsimonious than multinomial models [56].

For each outcome, we estimate separate models that include one of the three groups of key independent variables, and a full model that includes selected independent variables from all three groups. Model 1 includes all independent variables on general ecological conditions as well as personal conditions of production. Model 2 includes all independent variables on specific ecological conditions. Model 3 includes a selected subset of 6 variables on specific ecological conditions that are more directly related to climate change as well as personal conditions of production. Model 4 includes all independent variables on ownership of ecological conditions/natural resources (such as upland habitat acreage). Model 5 is the full model. We exclude additional control variables in Model 2 to preserve degrees of freedom. We exclude total cranberry acreage in Models 4 and 5 to avoid extreme multi-collinearity. Due to a constraint on the degrees of freedom posed by the small sample size, we are unable to include in the full models all independent variables, so we select independent variables based on the need for hypothesis testing, as well as the statistical significance tests of variables in prior, separate models. The sample size varies slightly across models due to the missing data for certain independent variables.

#### Communal conditions

Communal conditions are discussed in the context of our findings. Understanding the role that cranberry production plays in the Massachusetts cranberry growing region helps us discern the attitudes that growers hold, and gives us insights into the ways that the grower community approaches both their operation, but also issues associated with climate change. As discussed above, the communal conditions of cranberry production form a social network of tight-knitted growers and community members interested in seeing this industry survive in the Massachusetts landscape. Through site visits and in-depth interviews, the researchers recognize that growers are oftentimes very familiar with the bogs of neighboring growers, sometimes sharing equipment, water, and knowledge of local pests and other nuisances. Growers participate together in workshops held by the University of Massachusetts Cranberry Station, or the Cape Cod Cranberry Growers Association. Additionally, many growers participate in the annual cranberry harvest festival, and other events. The majority of growers are members of either the grower cooperative, Ocean Spray, or are connected to the private business, Decas, adding another level of networking to this agricultural production platform. Finally, cranberry growers are a rather small group, numbering around four hundred in total in Massachusetts, and they are clustered geographically in Southeastern Massachusetts.

## Results

### The cranberry grower sample population

We present the means and range of values for the dependent and independent variables in Table 2, pointing out some important characteristics of the sample population. The 91 cranberry growers who participated in the survey were mostly men older than 55 years of age. Generally, the population is less likely to see *global warming as a threat* (mean = 0.42, where 0 = no and 1 = yes). Growers are only a little worried about global warming (mean = 2.21 ±0.89 SD on a 1–4 scale where 0 = not worry at all and 4 = worry a great deal). Although, the majority of growers reported that conditions in general for cranberry production were getting worse (mean = 0.70 ±0.46 SD). Particularly noteworthy, growers report insect pests, weed pests, and disease as important environmental conditions (means > 4.0 on a scale of 1 to 5, where 1 = not important and 5 = very important). Growers on average manage 40.7 acres of cranberry bogs, with the minimum acreage of 1 acres and a maximum of 200. On average growers have access to 39.4 acres of wetland habitat and 102.6 acres of upland habitat. Thus on average for every acre of cranberry bog, growers manage 3.5 acres of natural habitat.

#### General attitudes

**Fig 3:**
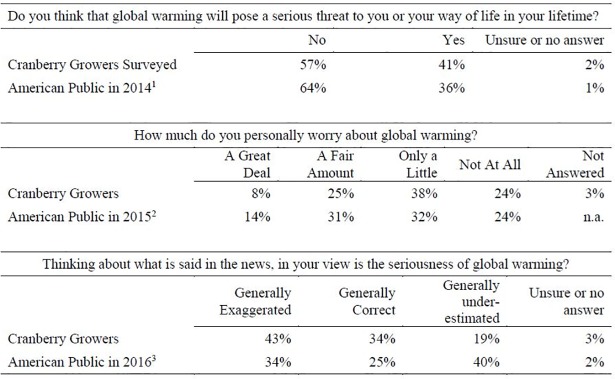
Comparing cranberry grower attitudes on global warming to the general american public. ^1^ http://www.gallup.com/poll/167879/not-global-warming-serious-threat.aspx; ^2,3^ http://www.pollingreport.com/enviro.htm.

Our data suggest that cranberry growers are more skeptical of the threat of global warming than the average American citizen (Fig 3). As we expected (Hypothesis I) cranberry growers are more likely to think that global warming will pose a serious threat to their way of life during their lifetimes than the general public (41% in comparison to 36%), but it is important to point out that 57% of cranberry growers do not think global warming is a threat to cranberry production. Contrary to our expectation, we found only 33% of the sample population reported feeling worried a fair amount to a great deal about global warming. In comparison 45% of the American public is worried about global warming. When asked how climate change is portrayed in the media, 43% of cranberry growers thought that global warming was “generally exaggerated” in comparison to 34% of the American public.

In sum, the majority of cranberry growers do not see global warming as threatening to their way of life and are generally not too worried about it, and slightly less than half think the seriousness of global warming is exaggerated in the media. Looked at another way, we find that the cranberry population is almost split down the middle between a group that recognizes the threat of climate change and is a little to very concerned about it and a group that does not view global warming as a threat and is not worried about it. To understand what predicts those differences, we look next to ecological and personal conditions of production.

#### General ecological conditions

Among the variables that measure general ecological conditions, *weather importance* and *weather event counts* are positively and consistently associated with *global warming threat* (Table 3). Based on Model 5, a one-unit increase (on a 5-point scale) of the reported importance of weather-related issues increases the odds of a grower considering global warming as a serious threat by 173.1% (2.731–1), holding other independent variables constant. Similarly, for each additional type of weather-related event a grower reports that severely affected their cranberry production, the odds of this grower considering global warming as a serious threat increases by 122.2% (2.222–1). In other words, growers who mention more weather-related events severely affecting their production, are much more likely to report global warming as a threat. These results support Hypothesis I, that growers reporting worsening ecological conditions will place greater importance on global warming.

**Table 3:** Odds ratios of logistic regression models of global warming threat: Cranberry growers of Massachusetts.

	Model 1	Model 2	Model 3	Model 4	Model 5
General ecological conditions					
General conditions (1 = getting worse, 0 = not getting worse)	1.435				2.852
Weather importance	2.230*				2.731*
Weather counts	2.026*				2.222*
Specific ecological conditions					
Water Scarcity		0.918	1.013		
Competition for Water		0.899	0.781		
Heavy Precipitation		1.171	1.172		
High Temperatures in Summer		0.736	0.658		
High Temperatures in Fall		1.725	1.743		
Insufficient Chill Hours in Winter		1.448	1.901#		1.353
Insect Pests		1.613			
Weed Pests		0.918			
Disease		0.861			
Access to ecological conditions					
Wetland habitat acreage (unit: 10 acres)				1.039	1.072
Upland habitat acreage (unit: 10 acres)				0.982	0.959
Relative size of natural habitat (% total farm size)				0.977	0.957#
Additional control variables: Personal conditions					
Education attainment	1.628*		1.730*	1.364	2.021*
Age group	0.594#		0.722	0.572*	0.523#
Total cranberry acreage	1.016		1.051		
Female	4.872*		2.316	2.439	5.327#
Constant	0.0016*	0.0456#	0.0084*	5.179	0.0016*
N	78	83	78	76	69
Pseudo R-squared	0.313	0.105	0.212	0.116	0.405

# p<0.1

* p < .05 (two-tailed tests)

*Global warming worry* was analyzed with parameterizations similar to the models in Table 3, the only difference being that the importance of high temperatures in summer is statistically significant in Models 2 and 3 and is thus added to Model 5 (the full model). In Models 1, 4, and 5, we report three odds ratio for the personal condition variable *female* because it violates the proportional odds assumption (see Table 4). The outcome *global warming worry* has 3 cut points: worry a little bit or more vs. not worry at all; worry a fair amount or a great deal vs. not worry at all or worry a little bit; worry a great deal vs. worry a fair amount or less. Variables that measure general ecological conditions of production are not associated with *global warming worry* in Model 1, although *general ecological conditions* and *weather counts* both become positively associated with *global warming worry* in Model 5. Odds of worrying more about global warming increases by 227.3% (3.273–1) for growers who think the general conditions for cranberry production are getting worse, holding other predictors constant. Similarly, each additional type of weather-related event a grower reports affecting cranberry production increases the odds of this grower worrying more about global warming by 47.5% (1.475–1), shown in Table 4.

**Table 4:** Odds ratios of generalized ordered logit regression models of global warming worry: Cranberry growers of Massachusetts.

	Model 1	Model 2	Model 3	Model 4	Model 5
General ecological conditions					
General conditions (1 = getting worse, 0 = not getting worse)	1.422				3.273*
Weather importance	1.274				1.203
Weather counts	1.169				1.475#
Specific ecological conditions					
Water Scarcity		0.825	0.987		
Competition for Water		1.185	1.058		
Heavy Precipitation		1.213	1.105		
High Temperatures in Summer		0.525#	0.433*		0.463#
High Temperatures in Fall		1.291	1.181		
Insufficient Chill Hours in Winter		1.547#	2.123**		1.947*
Insect Pests		1.174			
Weed Pests		1.203			
Disease		0.755			
Access to ecological resources					
Wetland habitat acreage (unit: 10 acres)				1.092*	1.152**
Upland habitat acreage (unit: 10 acres)				0.962*	0.944*
Relative size of natural habitat (% total farm size)				1.007	1.005
Additional control variables: Personal Conditions					
Education attainment	1.214		1.587*	1.080	1.499#
Age group	1.003		1.040	0.720	0.900
Total cranberry acreage (unit: 10 acres)	1.060		1.053		
Female	0.965^a^			0.924^a^	0.770^a^
	4.199*^b^		0.968	4.353*^b^	4.968*^b^
	1.161^c^			2.366^c^	2.561^c^
Constant	0.257^a^	2.659^a^	0.209^a^	7.890^a^	0.175^a^
	0.0196*^b^	0.322^b^	0.0196*^b^	0.581^b^	0.0077*^b^
	0.0052**^c^	0.0621#^c^	0.0036**^c^	0.121^c^	0.0016**^c^
N	79	84	79	77	69
Pseudo R-squared	0.0784	0.0475	0.0830	0.0646	0.1471

# p<0.1

* p < .05

** p < .01 (two-tailed tests)

Dependent variable coding: (1) not worry at all; (2) worry a little bit (3) worry a fair amount; (4) worry a great deal. For variables that violate the proportional odds assumption:

^a^ odds of worrying at least a little bit rather than not worrying at all

^b^ odds of worrying at least a fair amount rather than worrying a little bit or less

^c^ odds of worrying a great deal rather than worrying a fair amount or less.

#### Specific ecological conditions

The only specific environmental variables that are associated with *global warming worry* relate to temperature–the importance of high temperatures in summer and insufficient chill hours in winter, only partly supporting Hypothesis II. Based on Model 5, a one-unit increase (on a 5-point scale) in the reported importance of the insufficient chill hours in winter increases the odds of a grower considering global warming as a serious threat by to 90.1% (1.901–1) and worrying more about global warming by 94.7% (1.947–1). In contrast, a one-unit increase in the reported importance of high temperatures in summer reduces the odds of a grower worrying more about global warming by 53.7% (1–0.463). Said another way, growers who report worsening insufficient chill hours in winter are more likely to see global warming as a threat and worry more about it, while the opposite is observed for growers who report worsening high temperatures in summer (Table 4).

#### Ownership of ecological conditions

The results provide mixed evidence for Hypothesis III––*increased ownership of ecological conditions of production will have an inverse effect on attitudes towards the importance of global warming*. We found that cranberry growers who have access to larger amounts of natural habitat relative to their farm size are less likely to consider global warming as a serious threat (Model 5), such that a one percentage point increase in the relative size of natural habitats (% total farm size) reduces the odds of a grower considering global warming as a serious threat by 4.3% (i.e., 1–0.957) (Table 3). The relative size of natural habitats, however, is not associated with *global warming worry*. Looking more specifically at the types of habitat, we found that growers who have access to larger amounts of upland habitat (in actual acreage) are less likely to worry about global warming, supporting Hypothesis III. However, those who have access to larger amounts of wetland habitat are more likely to worry about global warming, which works against Hypothesis III. According to Model 5, a 10-acre increase in the size of wetland habitat that a grower can access increases the odds of this grower worrying more about global warming by 15.2% (1.152–1), as shown in Table 4. This suggests that access to specific natural resources and habitat, as opposed to their ownership in general, and farm size are important to consider and can have different effects.

#### Personal conditions of production

Consistent with extant literature, and Hypothesis IV, personal conditions of production affect global warming attitudes. We find that education, gender, and age have some effect, but not in all models. Higher level of education, up until baccalaureate attainment, is associated with greater odds of worrying more about global warming (Table 4). Growers with a Master’s degree express similar lack of concern to those with less than a baccalaureate degree. Female growers are more likely to view global warming as a threat and to worry about it a fair amount. Age group (being older) is associated with a lower odds ratio of reporting global warming as a threat (Table 3), and not associated with *global warming worry* (Table 4). Finally, total cranberry acreage is not associated with *global warming threat* or *global warming worry* (Table 4 and Fig 3).

## Discussion

Why is it that cranberry growers, who report that conditions are getting worse for cranberry production, are not particularly worried about global warming when their production system depends on ecological means of production in ways that are distinctive from other production systems? We anticipated that the highly integrated social and ecological system of cranberry production along with observed changes to the ecological conditions would override any conservative bias, which can downplay global warming as a serious threat. While we did not explicitly ask about political party affiliation or political leaning, growers’ answers to a survey question about their opinions on the seriousness of global warming portrayed in the media suggests that this population sample may also holds conservative views about global warming.

However, with almost half of the sample reporting some concern over global warming and the majority reporting some concern, if only a little, cranberry growers’ climate change attitudes are more nuanced than being described simply as “skeptical.” Our findings shed light on the complex relationship between personal, communal, and ecological conditions of cranberry production and the attitudes held of Massachusetts cranberry growers on climate change. Drawing from these conditions helps us understand the context in which cranberry growers are embedded, helping us discover the social and ecological situation that contributes to attitudes on global climate change. We find that some conditions create openings in which climate change discussions have meaning to cranberry livelihoods. For one, growers that report weather as an important condition are more likely to see global warming as a threat. Weather is a broad phenomenon that can be felt and described over time, from season to season and, culturally, from generation to generation.

It seems surprising that water scarcity and competition for water were not associated positively with global warming attitudes. However, New England is a region with a history of variable precipitation, such that cranberry growers have long dealt with deficits in precipitation as much as higher than normal precipitation. Furthermore, technologies to level fields, pump water, and drain bogs has made it easier for growers to manage water variability. We posit that cranberry bogs are one of the most resistant systems to drought and flooding, because they are naturally low in the landscape where water accumulates, the water table sits below a thick layer of water absorbent peat (one of the authors has walked on peat-based bogs that felt like walking on a water bed), and bogs have a system of ditches, levees, and gates to allow water to move through them and be released in times of flood. Additionally, communal conditions allow growers to share water resources, helping each other during drought times. In sum, while water is critical to the growth and harvest of cranberries, it is something that cranberry growers are accustomed to managing collectively under variable precipitation levels.

Temperature changes, however, are another matter for cranberry growers. Insufficient chill hours during winter were positively associated with *global warming threat* with good reason. Unlike drought or flooding, there is nothing cranberry growers can do for their plants to compensate for insufficient freezing temperatures. Although if cranberry plants break dormancy early in spring, growers manage the risk of frost damage by regularly irrigating the fields. Through in-depth interviews and observations, we understand from growers that this is arduous work as it entails periodically testing all the sprinkler heads throughout the night and dawn. As one grower said to us, “we’d prefer that the bogs stay covered in snow until the end of April” (personal communication), because then the plants, with more than sufficient chill hours met, break dormancy and develop without the constant threat of a damaging frost. Unfortunately opportunities for long cold winters are slipping away as winter months are getting warmer and more variable. For example, on 21 Feb 2018 the high temperature in Boston was 22.2°C at 12 pm; 24 hours later the temperature was 3.8°C–a difference of more than 18°C. If growers are to maintain their production amid warming temperatures, they will need to rely on communal conditions, on a community network of support, increased input from extension, the development of new cranberry varieties with lower chill hour requirements and frost tolerance, and likely increased support from the state.

We also discover that personal conditions of production have an effect on global warming attitudes, where being female, and to a lesser extent, being more educated, are both positively associated with the degree to which a person recognizes global warming as a serious threat and worries about it. We know from our observations that many cranberry growers are women, women who are responsible for all aspects of their cranberry growing operation, while a larger percentage of women are involved in cranberry production as a family business, from managing the books and labor to participating in the harvest. Women cranberry growers might thus play an important role in climate change adaptation and raising awareness among what is a relatively small community of cranberry growers. As we know, communal conditions of production involve both infrastructural and cultural connections that allow for economic activities to flourish, and this tight-knit community could learn from the knowledge of this sub-group. Education to the extent of receiving a bachelor's degree was the threshold for being more concerned about global warming. People with less education as well as people with graduate education showed less concern for global warming. This pattern concurs with McCright and Dunlap’s findings which demonstrated that white, well-educated males were most skeptical about climate change [32,55].

Besides the importance of chill hours the most revealing finding for us is the importance of the ownership of certain ecological conditions of production over others. While both wetland and upland habitat were associated with global warming attitudes, an increase in wetland habitat increases odds of worry while an increase in upland habitat reduces those odds. We suspect that this has to do with the intimate relationship that cranberry growers have to their surrounding landscape; the style of cranberry growing might be the reason for this finding. This meshes with scholarship in political ecology that identifies varying environmental attitudes, perceptions of ‘nature,’ and interest in working to resolve community-level environmental problems among small-scale farmers [46–48]. Cranberry growers are deeply dependent on water throughout the year to maintain a healthy harvest, making them likely to be much attuned to the effects that a warming region is having on its water supply. Additionally, drought and flooding can be seen in water levels, providing a visual cue that conditions are not average. It is possible that the growers who own wetland habitat have greater awareness of the effects on water, on their ability to use it when in need, even if they do not necessarily associate this with its impact on cranberry growing itself. On the other hand, simply having access to an abundance of dry upland habitat might act as a buffer to anxiety about a changing climate because owning land can be a powerful feeling. The upland habitat may be exploited to expand cranberry bog production or for other purposes, such as timber. However, we stress that we are speculating here and would suggest further research on these intriguing trends should follow.

## Conclusion

Massachusetts cranberry bogs are particularly vulnerable to climate change impacts due to their high chill hours requirements, perennial habit (making them harder to re-establish in a new bog or rotate with other crops for pest control), and their high dependence on great quantities of water. However, we found that cranberry growers, with the exception of women, are generally less concerned about global warming than the average American, despite the fact that increasing temperatures and more variable precipitation are predicted for the region. We suggest two main reasons for this trend: one that growers may lean more politically conservative than the average American, as is typical of many American farmers [7,30]; and second, that cranberry growers have always dealt with a variable and challenging climate in New England. As a grower told one of us, “this year it’s drought, next year it’ll be too much water” (personal communication). Thus, lack of concern must be taken in the context of their overall concern in dealing with the elements of weather. Because of the annual production cycle of cranberries (managing frosts in the spring, pests in the summer, water for wet harvest in the fall), growers are likely too absorbed in the challenges of the growing cycle to be concerned about long-term climatic changes. Somehow they have always managed to get by and in fact, the average cranberry yield per acre has been increasing due to the introduction of new high yielding varieties from Rutgers University (See Rutgers Licensing and Technology: Agricultural Products website, http://agproducts.rutgers.edu/cranberries/varieties.html.). However, when global warming reduces the number of chill hours accumulated during the winter below the requirements for successful cranberry bud development, it is likely that cranberry growers will experience a novel growing environment and cranberry production in Massachusetts will take a big economic hit. Perhaps then concern over global warming will increase among all cranberry growers. Thus, personal, communal and ecological conditions of cranberry production have distinct effects on how cranberry growers approach both their cranberry growing operation and broader ecological changes impacting the world. What is worth further investigation in particular is the finding that women cranberry farmers, who tend to be more worried about global warming, might be important agents for community adaptation to a warming New England.

Our study, the first of its kind on cranberry production, suggests that ecological conditions also matter a great deal. Cranberry growers’ attitudes on climate change are shaped in part by the ecological landscape in which they are enmeshed. This finding is particularly important because cranberry growers on average manage more than three times as much upland and wetland habitat than acres of cranberry bog. The cranberry production landscape is a mosaic of peaty cranberry bogs, man-made cranberry bogs, forest, meadow, ponds, streams, rivers, and suburban habitat, and it turns out that the amount of upland and wetland habitat a grower has makes a difference in the grower’s concern over climate change. Growers will need to take advantage of its tight-knit communal conditions in order to attempt to sustain this production system in a warming New England.
